# Supplementary material for: Tools and Biomarkers for the Study of Retinal Ganglion Cell Degeneration
Source: Int J Mol Sci. 2022 Apr 13;23(8):4287. doi: 10.3390/ijms23084287 (PMC9025234; doi:10.3390/ijms23084287)
Supplement: Supplementary file 1 [file ijms-23-04287-s001.zip › ijms-1651879-supplementary.pdf]

**SUPPLEMENTARY INFORMATION**

Table S1: **RGC Classification.** The table shows the different subtypes of ganglion cells based on the standardized type definitions from Gregory W. Schwartz's research group at Northwestern University (USA) [26,27]. They have created a resource of physiological, morphological, and transcriptomic data aimed at establishing a comprehensive typology of mouse RGCs (http://rgctypes.org/). Otherwise when data came from different source or are referred to subtypes not included by this authors, it is indicated in the table

| Cell type | Cell subtype | Morphology features | Physiology features |
|---|---|---|---|
| **ON-OFF Directionally Selective Ganglion Cells** | OODS D | Somas of around 16 μm diameter.<br>Dendritic arbors of about 200 μm with ON and OFF stratification with an asymmetric shape differently oriented for each case.[1–4] | Heightened sensibility to non-stationary stimuli which move in a specific direction (DSIs between 0.3 and 0.5 depending on the response and type). Transient ON and OFF responses with short latencies. ON RFs of 130 μm for S and I ooDSG ON and OFF RFs of 200 μm for the T and N ooDSG. [1,2,4] |
| | OODS T | | |
| | OODS V | | |
| | OODS N | | |
| | OODS Direction unkown | | |
| **ON Directionally Selective** | ONDS sustained Dorsonasal | Their dendritic arbors stratify in the ON layer of the IPL have a diameter of about 300 μm. SC-projecting ON-DSGCs have a more symmetric and smaller dendritic arbor area than MTN- projecting ON-DSGCs.[4,5] | Heightened sensibility to ON non-stationary stimuli of their preferred direction (DSI 0.60) ON sustained responses with short latencies except for the transient type. RF are around 320 μm.[4,6] |
| | ONDS sustained Temporal | | |
| | ONDS sustained Ventral | | |
| | ONDS sustained Direction unknow | | |
| | ONDS transient [6] | | |
| **Orientation Selective** | ON vertical OS small RF (a*) | Bistratified; ON layer proximal to the inner ChAT band and OFF layer distal to the outer ChAT band | Preferential responses to stimuli presented in a certain axis of the visual field. For ON cells, a full field stimulus elicits a very characteristic response consisting of a short burst of spikes which is followed by a period of silence after which the main response occurs. For OFF cells, this silence period does not exist and instead a small intensity sustained response is elicited. Orientation selective stimuli do not provoke this short pause. Small field types show a decreased response to stimuli over 200-250 μm while the responses of the complementary types start reaching their maximums at that size and maintain intensity regardless of size increase. [7] |
| | ON vertical OS large RF (a *) | | |
| | ON horizontal OS small RF (a *) | ON hOS RGC diameter: ON~338 μm OFF ~ 239 μm | |
| | ON horizontal OS large RF (a *) | ON vOS RGC diameter: ON ~ 252 μm OFF ~195 μm.[7] | |
| | OFF horizontal OS | Symmetric dendritic arbor with a diameter of about 200 μm, which stratifies in a band exterior to the OFF ChAT and exterior to the OFFvOS. | |
| | OFF vertical OS | Asymmetric dendritic arbor with all dendrites arborizing in one side of the soma which stratifies in a band | |

| | | | |
|---|---|---|---|
| | | exterior to the OFF ChAT. | |
| **Intrinsically Photosensitive Melanopsin-Containing Retinal Ganglion Cells** | M1 | Somas with a diameter of 13-17 μm and big dendritic fields (275- 350 μm) but with few primary dendrites and little branching, stratifying in a narrow band adjacent to the inner nuclear layer and forming *en passant* synapses with Type 6 ON bipolar cells.[8–13] | When it comes to ipRGCs many authors make a differentiation between the intrinsic responses which derive from the RGCs own opsins and extrinsic responses which are derived from the input of rods and cones. As a generalization, the intrinsic responses of M1 cells are faster, of higher amplitude and have a lower threshold. M2 and M3 ipRGs have bigger responses than M4 and M5 cells with M1 responses in between. M6 responses cannot be fully compared with published data a because of differences in the intensity of the light stimuli or the melanopsin allele number in experiments, but are weakly intrinsically photosensitive. In *Opn4*$^{-/-}$ mice M2 and M3 ipRGCs have light responses similar to those of wild-type animals while M1 ipRGCs have reduced responses. This is probably due to the fact that M1 cells normally function due to intrinsic input and M2 and M3 cells function due to extrinsic input. Responses to light steps from darkness of ~200 R*/rod/s without suppressing either pathway. M1 RGCs responds with shorts bursts of spikes interspaced with periods of silence. Spike frequency decays during the duration of the stimulus from an initial peak of medium (~0.2s) latency. The M2 type show an ON sustained response with a short latency while the M6 shows an ON transient response with also a short latency. Receptive fields have also been characterized by several authors sometimes with different results. M1 and M2 cells as being optimized to answer to stimuli of 400 μm with little changes with |
| | M2 | Big somas (15-22 μm) and big dendritic fields (314- 425 μm) stratifying in the ON sublamina, vitreal to the ON ChAT band, vitreal to the M4 dendrites with limits at 67%± 0.06% and 83% ± 0.03% depth. Viney et al place their dendrites at a depth of 30% ± 4 which corresponds to the S4-5 layers of the IPL. [6,8–10,13,20] | |
| | M3 | Big somas (17 μm) and two very big dendritic arbors which stratify in the ON and OFF sublaminae (with a variable proportion on each).[9] | |
| | M4 (ON sustained in some publications) | Same as alpha ON. Big somas (20 μm) and big dendritic arbors (300-370 μm). Changes with the nasal-temporal axis; with arbors growing up to 100 μm in the nasal locations [114]. The arbors stratify in the ON sublamina vitreal to the ON ChAT band, above/distal to the M2 dendrites. Krieger et al described the ON-T in the inner part of first quartile and the ON-S in the outer part of first quartile.[12,14,21] | |
| | M5 | Somas of around 14 μm. Dentritic arbor 224 ± 44 μm, stratifies in the ON sublamina. [12,17] | |

| | | | |
|---|---|---|---|
| | M6 | Somas of about 13 μm with two dendritic arbors which stratify in the ON and OFF sublaminae. The ON arbor stratifies below but proximal to the ON ChAT band while the OFF arbor stratifies narrowly in the distal extreme of the OFF sublamina (the ON arbor accounts for 84 ± 12% of the total dendritic length). The dendritic field diameter is about 215 μm. [15] | bigger spots and M6 having its optimization zone between 100 and 350-400 μm. Other authors establish the RF of ipRGCs as M1-M4~ 200 μm M5 165 μm M6 390 μm. The M1 ipRGC receptive field has no perceptible surround effect due to the offset effect that the surround of the amacrine input has on the bipolar input and vice versa. The rest of ipRGC show a center surround receptive field. M5 and M4 also show chromatic opponency iPRGCs do not display directional sensitivity, but M1 M2, M3 and M4 ipRGCs have been demonstrated to display a preferential response for stimuli of a certain speed.[8,14–19] |
| **Small field** | LED | A dendritic arbor of 131 ± 6 μm of diameter which stratifies between the ON and OFF ChATs (66 ± 2%).[22] | Small field RGCs are a group of ganglion cells which show both ON and OFF responses of differing characteristics to small stimulus. LED RGCs show ON dominant responses in scotopic conditions, OFF dominant responses in photopic conditions and both their ON and OFF responses were completely suppressed for spots ~ 300 OFF in scotopic and ON photopic and ~ 400 ON in scotopic and OFF photopic. LEDs have sustained responses (mean AP of 0.8s, all HD RGCs have shorter AP than 0.6s) and long latencies (0.4s) LEDs are object motion sensitive. HD1 cells show stronger ON responses than OFF responses both in scotopic and photopic conditions. Responses are transient (mean AP of 0.4s) with short latencies (0.15s) HD1 RGC are object motion sensitive. HD2 show stronger OFF responses than ON responses both in scotopic and photopic conditions. OFF responses of HD2 RGCs were completely suppressed at spot sizes 400 μm, but ON responses were never fully suppressed even at spots sizes up |
| | UHD | A dendritic arbor of 99 ± 7 μm of diameter which stratifies between the ON and OFF ChATs (59 ± 5%).[22] | |
| | HD1 | A dendritic arbor of 144 ± 5 μm of diameter which stratifies between the ON and OFF ChATs (60 ± 3%).[22] | |
| | HD2 | A dendritic arbor of 143 ± 6 μm of diameter which stratifies between the ON and OFF ChATs (59 ± 2%).[22] | |

| | | | |
|---|---|---|---|
| | | | to 1200 μm. Responses are transient (mean AP shorter than 0.2s) and have short latencies. UHD RGCs display equivalent spike rates at light onset and offset and are completely suppressed for spots > 275 μm in scotopic conditions and were OFF dominant in photopic conditions. Responses are transient (mean AP shorter than 0.2s) and have short latencies.[4] |
| **Alpha RGC** | OFF sustained<br>OFF transient<br>ON (b*) | Big somas (>15 μm OFF-T cells ~20–25 μm) and big a mono-stratified dendritic arbor (OFF; 250-300 μm ON; 210 μm). The level of stratification depends on the type with the ON type stratifying in a band vitreal to the ON ChAT (Krieger et al described the ON-T in the inner part of first quartile and the ON-S in the outer part of first quartile), the OFF-T stratify between the ChATs just next to the OFF band (third quartile/30%–35% ) and the OFF-S outside the OFF ChAT next to the outer IPL border , in the fourth quartile.[21,23,24] | Short response latency (around 60ms and similar across types). The ON type shows sustained responses. The OFF- T has a decay time of ~50ms while the OFF-S of ~250 ms. No directional preference, however OFF-T cells show a preferential response to approaching stimuli. RF of 200-250 μm.[21] |
| **Suppressed by contrast RGCs** | Bursty SbC | Dendritic arbor of 200 μm diameter with a stratification in a band exterior to the OFF ChAT (from 1.2 to 1.5 normalized). | OFF responses with short latency to stimuli of any sizes. |
| | Sustained SbC EW27 | Dendritic arbor of 200 μm diameter, stratification in two small bands exterior to the ON and OFF ChATs. | Responses to OFF stimuli 140 μm and bigger with a short intense response and a longer maintained stimuli. |
| | Sustained SbC EW28 | Asymmetric arbor shape, area of 37.500 μm, stratification in two bands exterior to the ON and OFF ChATs. | Transient ON and more sustained OFF responses with stimuli up to 400 μm. ON sustained responses of longer latency to stimuli bigger than 500 μm. |
| **FOXP2 Positive RGC** | F-mini ON | Small dendritic arbor with an area of around 15000 μm which are asymmetric towards ventral pole in the dorsal retinal and to the | Transient ON responses with a short latency with a maximum response to stimuli of around 150 μm and a RF of 66 ± 4 μm [73] Directionally selective DSI: 0.33 ± |

| | | | |
|---|---|---|---|
| | | dorsal pole in the ventral retina. The polarity changes in the S to M transition zone. The directional selectivity depends on the direction of the arbor asymmetry. Stratification at ~ SL3 in a thick band between the ChATs.[25] | 0.04 (to the direction of the RGC and according to their polarity).[25] |
| | F-mini OFF | Small dendritic arbor with an area of around 15000 μm with an asymmetry towards the ventral pole. Stratification at ~ SL1 external to the OFF band.[25] | Transient OFF responses with a short latency with a maximum response to stimuli of around 170 μm and a RF of 66 ± 4 μm [73]. Directionally selective DSI: 0.33 ± 0.04 (to the direction of the RGC and according to their polarity). [25] |
| | F-midi ON (c*) | Asymmetry towards the ventral pole. ~ SL3. | RF of 85 ± 8 μm. Not directionally selective. [25] |
| | F-midi OFF (c*) | Asymmetry towards the ventral pole. ~ SL1.[25] | RF of 85 ± 8 μm. Not directionally selective.[25] |
| **Non Grouped/ON transient** | ON transient EW6t | Small (about 200 μm or less of diameter), dense, asymmetric arbor which stratifies between the ChATs just interior to the ON ChAT. | Transient small short latency ON responses optimized for stimuli around 150 μm. |
| **Non Grouped/ON sustained** | PixON | Asymmetric arbor shape with an approximate area of 45.000. Stratification vitreal to the ON ChAT band ( -0.65 normalized) | Sustained short latency ON responses to stimuli from 100 to 400 μm. |
| **Non Grouped/OFF sustained** | OFF sustained EW3o | Dendritic arbor stratifying in a band just outside the OFF ChAT with a diameter of around 200 μm. | Sustained short latency OFF responses to stimuli optimized at a sized over 200 μm. |
| **Non Grouped/OFF sustained** | OFF sustained EW1no | Dendritic arbor stratification in a band outside the OFF ChAT (Around 2 Normalized depth) with a diameter of around 225 μm. | OFF sustained responses with a short latency to stimuli over 50 μm and optimized at 200 μm |

| | | | |
|---|---|---|---|
| **Non Grouped/OFF transient** | OFF tr MeRF | Medium dendritic arbor (about 22000 μm of area) which stratifies just vitreal to the OFF Chat. | OFF transient responses with short latencies optimized for stimuli around 150 μm. |
| **Non Grouped/OFF transient** | OFF tr SmRF | Medium (about 150 μm of diameter) dendritic arbor which stratifies just vitreal to the OFF Chat. | OFF transient responses with short latencies optimized for stimuli around 250 μm. |
| **Non Grouped/ OFF sustained** | OFF medium sustained | Dendritic arbor stratification in a band outside the OFF ChAT just adjacent to it with an area of 35000 μm. | OFF response with an intermedium decay of the signal, a short latency and a small response to ON stimuli. Optimized to stimuli of about 100 μm and bigger. |
| **Non Grouped/ ON transient** | ON transient small RF | Dendritic arbor stratification in a band outside the ON ChAT just adjacent to it with a diameter of 180 μm. | ON transient responses with a short latency to stimuli which are optimized at a size of around 200 μm with spike count decreasing for smaller and bigger stimuli. |
| **Non Grouped/ ON transient** | ON transient Medium RF | Dendritic arbor stratification in a band outside the ON ChAT just adjacent to it with a diameter of 210 μm (area of 35000 μm). | ON transient responses with a short latency to stimuli which are optimized at a size of over 200 μm. However bigger stimuli still elicit responses. |
| **Non grouped/other** | ON small OFF large | Dendritic arbor stratification in a band outside the OFF ChAT with a diameter of 195 μm | Responses to ON stimuli of around 100 μm and OFF stimuli of 100 μm or bigger. Responses suffer an intermedium decay of the signal and the latency is short. |
| **Non grouped/other** | ON bursty | Stratification in the ON ChAT (Around 1 Normalized depth) with a diameter of 225 μm | ON sustained responses with short latency (0.14s) and a preferential response to spots around 180 μm diameter. |
| **Non grouped/other** | ON delayed | Area of 30.000 μm, stratification predominantly in the ON ChAT with a minor arbor in the OFF ChAT | ON sustained responses with a long latency (0.4s) to responses from around 140 μm diameter. |
| **Non grouped/other** | Motion Sensor | Big dendritic arbor of more than 400 μm of diameter stratifying between the ChATs. | When tested with flashed spots of light (200 R*/rod/s) from darkness motion sensor cells respond with a small ON transient and long latency response at light on and a small OFF transient and short |

| | |
|---|---|
| | latency response at light off. |

(a*) Most literature divide ON OS RGC into only two types which are characterized by their preferential responses to stimuli in the horizontal (nasal, temporal) and vertical (dorsal, ventral) axis respectively [4,7,28]. Gregory W. Schwartz classification further subdivides these types into four types according to the size of their receptive field ( ). Baden et al [29] also describes several clusters which contained around 30% of orientation sensitive cells. G14 cells had an ON-OFF polarity and were selective for vertical and horizontal orientations. G17 cells had an ON polarity and had orientation selectivity for directions which could vary around the 360º. Other clusters which displayed orientation selectivity were the G1 and the G30.

(b*) Krieger et al [21] describe two types of ON alpha RGC the transient and sustained types. However, they fail to find a mosaic for the ON-Transient type and other classifications such as the one proposed by Gregory W. Schwartz's group (upon which we base our table) (http://rgctypes.org/) only include the ON-Sustained type as the ON alpha type.

(c*) Rousso et al [25] describe four types of RGC expressing the Foxp2 protein. They are comprised of two pairs of small fields RGC which act in a complementary manner. The F-midi pair was not identified as a differentiated natural type in other classifications such as the one proposed by Gregory W. Schwartz's (upon which we base our table). This pair was probably divided into other types, but they remain a useful category as they are easily marked by surface antibodies.

OODS: ON-OFF Directionally Selective ooDSG: ON-OFF Directionally Selective Ganglion Cells DSGCs: Directionally Selective Ganglion Cells iPRGCs: intrinsically Photosensitive Retinal Ganglion Cells M1-M6: melanopsinic RGC hOS horizontal Orientation-selective vOS vertical Orientation-selective HD: High definition, UHD: ultra-high definition, LED: Local edge detector D: Dorsal T: Temporal V: Ventral N: Nasal DN: Dorsonasal DSI: Direction Sensitivity Index RF: Receptive Field DS: Directionally Selective SC: superior Colliculus MTN: medial terminal nucleus ChAT: Choline Acetyltransferase AP: Action Potentials SbC: supressed by contrast F-mini/midi : FOXP2 Positive mini/midi RGC S: S cones M M cones S : Also Superior equivalent to dorsal Inferior equivalent to ventral PixON: Pixel Encoder RGC tr: transient.

Table S2: **Transgenic lines for RGC labelling.** Transgenic mice alone or in combination with virus infections are used to label RGC in general or specific subtypes.

| Mouse Line | Cell type | Reference |
|---|---|---|
| **Cart-Tg1-Cre** | Population probably enriched for ooDSGC (tested by dendrite stratification and by CART and Pvalb antibodies) | [3,30] |
| **Cdh6-CreER** | Population probably enriched for ooDSGC (tested by dendrite stratification and by CART and Pvalb antibodies) | [3,30] |
| **Cnnm2-Cre_KO250** | Population probably enriched for ooDSGC (tested by dendrite stratification and by CART and Pvalb antibodies) | [30] |
| **Gpr26-Cre_KO250** | Population probably enriched for ooDSGC (tested by dendrite stratification and by CART and Pvalb antibodies) | [30,31] |
| **Hb9:eGFP** | A/T-ooDSGC | [2] |
| **Hoxd10-GFP** | N/P-ooDSGC and ON-DSGC | [4] |
| **TRHR-GFP** | Subtype of N/P-ooDSGC with a bigger receptive field (more angles of circle) and a symmetric dendritic arbor | [13] |
| **DRD4 GFP** | Subtype of N/P-ooDSGC with a smaller receptive field (less angles of circle) and slightly asymmetric dendritic arbor | [13,24] |
| **W9-RGCs** | Probably corresponds to same population (or similar) as DRD4. Positives for matrix metalloprotease 17 (Mmp17) | [3] |
| **FSTL4-Cre ER/TSY (BD-RGCs)** | Labels mostly I-ooDSGC and some S-ooDSGC. There are three different lines described by three groups which might differ due to the ectopic expression FSTL4-CreER caused by the integration in a different locus of the transgene. More than 90% of the labeled cells show an inferior preferred response and have a dendritic arbor with an inferior asymmetry. Around 10 % show superior preferred direction and have a dendritic arbor with a superior asymmetry.<br><br>Positive for cadherin 6 (Cdh6) and collagen 25a1 (Col25a1) | [1,3,32] |
| **Pcdh9-Cre_NP276** | ON-DSGC | [30,31] |
| **Grik4-Cre** | ON-DSGC<br><br>Some CART-positive ooDSGCs | [30,31] |
| **SPIG1-GFP/Fstl4** | D-ON DSGC (while SPIG1-GFP negative corresponds to V-ON DSGC) | [5,32] |

| | | |
|---|---|---|
| **Kcng4-Cre** | Labels all subtypes of Alpha RGCs | [21,30,33] |
| **TYW7-YFP** | Off-Alpha RGCs | [1,21] |
| **CB2-GFP** | Off-transient-alpha RGCs | [21] |
| **Calretinin-EGFP** | Off-transient-Alpha RGCs<br>Amacrine cells | [24] |
| **Crh-IRES-Cre** | OFF-layer stratifying Alpha RGC | [30,31] |
| **Etv1-CreERT2** | OFF-layer stratifying Alpha RGC | [30,31] |
| **Gal-Cre_KI87** | OFF-layer stratifying Alpha RGC | [30,31] |
| **CCK-**IRES-**Cre / rAAV2(YF4)-CBA-DIO-TVA - EnvA-SADΔG-GFP** | CCK 1 (70%) Described by Zhu et al. as similar to s-BGCs but for Ivanova et al. there is no partial overlap with ChATs. Possibly corresponds to SbC-RGC.<br>CCK 2 (20%) ooDSGC; one subpopulation has symmetric ON and OFF arbors. The other subpopulation has a ventrally asymmetric OFF arbor, which is similar to BD-RGCs and Hb9+ DSGCs .<br>CCK 3 (10%) ON-$\alpha$RGCs | [2,3,21,23, 33–35] |
| **Cdh3-GFP** | Mostly M6- ipRGCs, some M5- ipRGCs | [15] |
| **Opn4$^{cre/+}$** | Most sensitive line to label ipRGCs when crossed with the Z/EG or Z/AP reporter lines. 90% of labelled RGC are ipRGC, also labels cones and rods .<br>More unspecific when crossed with other reporter lines such as Ai9 or R26iAP [146]<br>Labels the axonal terminal of ipRGCs when crossed to the synaptophysin–tdTomato line | [15,37–39] |
| **Opn4-GFP** | M1–M3 in the adult mouse<br>M4 cells prior to P14<br>There are two lines; one has the GFP BAC transgene inserted between an upstream and a downstream region of the melanopsin locus [141] and the other has it inserted wholly upstream leaving the gene intact [142] | [40,41] |
| **Opn4-tdTomato** | M1, M2, and M4 iPRGCs [144] | [42,43] |
| **Opn4-tau-lacZ$^{+/+}$** | Melanopsin knockout ipRGC<br>M1 ipRGCs are beta-galactosidase positive in this line | [16,44] |
| **Opn4$^{DTA}$** | Ablates M1 to M3 cells by expressing the diphtheria toxin in the melanopsin locus | |

| | | |
|---|---|---|
| **Slc17a6-IRES-Cre** | Probably all RGC | [30,31] |
| **Thy1-Cre** | Probably all RGC | [30] |
| **Cux2-IRES-Cre** | Probably most RGC | [30,31] |
| **Drd1a-Cre** | Probably most RGC | [30,31] |
| **Jam2-Cre (not Jam2-CreER)** | Probably most RGC | [30] |
| **Htr2a-Cre_KM207** | Probably most RGC, but not DSGC (CART- and Foxp2-) nor ipRGCs (Opn4-). | [30,31] |
| **Calb2-IRES-Cre** | Probably multiple RGC types but not alpha-RGC (OPN-) | [30,31] |
| **Slc18a2-Cre_OZ14** | A subtype with stratification restricted to S2 and S4, not alpha-RGC (OPN-) | [30,31] |
| **FoxP2-IRES-Cre** | Probably multiple RGC but not alpha-RGC (OPN-) nor oo-DSCG (CART-) | [30] |
| **Jam2-CreER** | J-RGC (Stratifies in S1) | [30,45] |
| **Pcp2-Cre/GFP** | m-BGCs, s-BGCs, b-BGCs, on-DSGCs, ooDSGCs and bipolar cells | [35,46] |
| **Pvalb Cre × Thy1 Stp-EYFP** | PV1 to PV7 (types named according to their stratification depth) PV5 could be Alpha OFF-T subset, PV7 could correspond to the F-RGC OFF subtypes | [25,47] |
| **TYW3: W3 RGC** | LEDs and other populations (probably Small-Receptive-Field Ganglion Cells)<br>They predominate in the ventral retina. | [1,22,48] |
| **TYW7: W7 RGC** | Two morphological populations with the same physiological characteristics.<br>They predominate in the nasal retina. | [1] |
| **CRH-IRES-Cre/ *Thy1-STOP-YFP*** | Three subtypes CRH1: similar to W7 CRH2 and 3: similar to J-RGC | [34] |
| **Foxp2-Cre** | Expresses Cre in all F-RGC | [25,30] |
| **Pcp2-cre/GFP (Tg(Pcp2-cre)1Amc/J (Pcp2-Cre)** | Expresses Cre in all Purkinje positive RGC | [35] |
| **CCK-IRES-Cre/rAAV2(YF4)-CBA-DIO-TVA - EnvA-SADΔG-GFP** | Expresses Cre in CCK-1 RGCs among others | [30,34] |
| **Rbp4-Cre x Ai14 o Rbp4-Cre x AAV mCherry (Rbp4-Cre)** | R-RGC and RDS-RGC among others | [49] |
| ***$Ret^{CreERt2/WT}$; $ROSA26^{AP/WT}$*** | ON-alpha RGC, OFF alpha- RGC, OFF-beta RGC, ON beta RGC, ooDSGC, a small bistratified GC, and others | [50] |

| | | |
|---|---|---|
| ***Ret*$^{CreERt2/WT}$*; Brn3a*$^{CKOAP/WT}$ (Ret+/Brn3a+)** | OoDSGC, ON-snipy RGC, a small bistratified GC, OFF-Beta RGC | [50] |
| ***Ret*$^{CreERt2/WT}$*; Brn3b*$^{CKOAP/WT}$ (Ret+/Brn3b+)** | ON- beta, ON-alpha, OFF, alpha, OFF-DS, a big bistratified GC, unidentified bistratified RGC | [50] |
| ***Ret*$^{CreERt2/WT}$*; Brn3c*$^{CKOAP/WT}$ (Ret+/Brn3c+)** | On- snipy- RGC | [50] |
| **Brn3cCre/WT; ROSA26tdTomato** | Morphological: Two monostratified ON types, two monostratified OFF types, ooDSGC, a small bistratified GC, a bistratified GC with recursive dendrites<br>Physiological: ON-OS, OFF hOS, ON-OFF DS, On transient medium receptive field, | [51] |
| **Brn3cCre/WT; AAV2-CAG-FLEX EGFP** | ON-OS, OFF hOS, ON-OFF DS, HD1, ON transient small receptive field | [51] |

Abbreviations: Cre: recombinase cre for site specific recombination, GFP: Green Fluorescent Protein, EGFP: Enhanced Green Fluorescent Protein, YFP: Yellow fluorescent protein, EYFP: Enhanced Yellow fluorescent protein IRES: Internal ribosome entry site, OODS: ON-OFF Directionally Selective, ooDSG: ON-OFF Directionally Selective Ganglion Cells DSGCs: Directionally Selective Ganglion Cells, iPRGCs: intrinsically Photosensitive Retinal Ganglion Cells, M1-M6: melanopsinic RGC, hOS horizontal Orientation-selective, vOS vertical Orientation-selective, HD: High definition, UHD: ultra high definition, LED: Local edge detector, D: Dorsal T: Temporal V: Ventral N: Nasal, DN: Dorsonasal, DSI: Direction Sensitivity Index, RF: Receptive Field, DS: Directionally Selective, SC: superior Colliculus, MTN: medial terminal nucleus, ChAT: Choline Acetyltransferase, AP: Action Potentials, SbC: supressed by contrast, F-mini/midi : FOXP2 Positive mini/midi RGC, S: S cones, M: M cones, S : Also Superior equivalent to dorsal, Inferior equivalent to ventral, PixON: Pixel Encoder RGC, tr: transient S-BGC, M-BGC and B-BGC: Small, Medium, and Big Bistratified RG, RDS-RGC R Direction selective RGC, CART: cocaine- and amphetamine-regulated transcript, PV: Paralbumin, DRD4 Dopamine receptor D4, MMP17: Matrix metalloproteinase-17,Cdh3: Cadherin 3 ,Cdh6: cadherin 6, Col25a1: collagen 25a1, ChAT: Choline Acetyltransferase, CCK1,2,3: Cholecistokinin receptor 1,2,3; Opn4: Opsin 4 or melanopsin, S1-5: sublaminae of the IPL, IPL: inner plexiform Layer, Brn3b: POU domain, class 4, transcription factor 2,Brn3a: POU domain, class 4, transcription factor 1,Brn3c: POU domain, class 4, transcription factor 3,Kcng4: Potassium voltage dependent channel subunit, TRHR: thyrotropin-releasing hormone receptor, CB2: cannabinoid receptor 2, Tg1: transgenic line 1 Cdh6 cadherin 6 Cnnm2: Cyclin And CBS Domain Divalent Metal Cation Transport Mediator 2, Gpr26: G Protein-Coupled Receptor 26, Hb9: Motor neuron and pancreas homeobox 1 (MNX1), also known as Homeobox HB9, Hoxd10: Homeobox D10, TRHT: Thyrotropin Releasing Hormone Receptor, FSTL4: Follistatin Like 4, CreER: tamoxifen-responsive cre recombinase TSY: Thy1-STOP-YFP mice line 15, Thy1: Thy-1 Cell Surface Antigen, Pcdh9: Protocadherin 9, Grik4: Glutamate Ionotropic Receptor Kainate Type Subunit 4, SPIG1: SPARC-related protein-containing immunoglobulin domains 1, TYW7: Tg(Thy1-YFP)W7Jrs, CB2: cannabinoid receptor type 2, CRH: Corticoid Releasing hormone / Corticotropin Releasing Hormone , ETV1: ETS Variant Transcription Factor 1, ERT2: Estrogen

ligand-binding domain, Gal: Galanin And GMAP Prepropeptide, rAAV2: Recombinant adeno-associated virus 2, Slc17a6: Solute Carrier Family 17 Member 6, Cux2: Cut Like Homeobox 2, Drd1: Dopamine receptor D1, Jam2: Junctional Adhesion Molecule 2, Htr2a: 5-Hydroxytryptamine Receptor 2A, Calb2: Calretinin, also known as calbindin 2, Slc18a2: Solute Carrier Family 18 Member A2, FoxP2: Forkhead box protein P2, Pcp2: Purkinje Cell Protein 2, Rbp4: Retinol Binding Protein 4

## References


1. Kim, I.-J.; Zhang, Y.; Meister, M.; Sanes, J.R. Laminar Restriction of Retinal Ganglion Cell Dendrites and Axons: Subtype-Specific Developmental Patterns Revealed with Transgenic Markers. *J. Neurosci. Off. J. Soc. Neurosci.* **2010**, *30*, 1452–1462, doi:10.1523/JNEUROSCI.4779-09.2010.
2. Trenholm, S.; Johnson, K.; Li, X.; Smith, R.G.; Awatramani, G.B. Parallel Mechanisms Encode Direction in the Retina. *Neuron* **2011**, *71*, 683–694, doi:10.1016/j.neuron.2011.06.020.
3. Kay, J.N.; De la Huerta, I.; Kim, I.-J.; Zhang, Y.; Yamagata, M.; Chu, M.W.; Meister, M.; Sanes, J.R. Retinal Ganglion Cells with Distinct Directional Preferences Differ in Molecular Identity, Structure, and Central Projections. *J. Neurosci. Off. J. Soc. Neurosci.* **2011**, *31*, 7753–7762, doi:10.1523/JNEUROSCI.0907-11.2011.
4. Dhande, O.S.; Estevez, M.E.; Quattrochi, L.E.; El-Danaf, R.N.; Nguyen, P.L.; Berson, D.M.; Huberman, A.D. Genetic Dissection of Retinal Inputs to Brainstem Nuclei Controlling Image Stabilization. *J. Neurosci. Off. J. Soc. Neurosci.* **2013**, *33*, 17797–17813, doi:10.1523/JNEUROSCI.2778-13.2013.
5. Yonehara, K.; Ishikane, H.; Sakuta, H.; Shintani, T.; Nakamura-Yonehara, K.; Kamiji, N.L.; Usui, S.; Noda, M. Identification of Retinal Ganglion Cells and Their Projections Involved in Central Transmission of Information about Upward and Downward Image Motion. *PloS One* **2009**, *4*, e4320, doi:10.1371/journal.pone.0004320.
6. Gauvain, G.; Murphy, G.J. Projection-Specific Characteristics of Retinal Input to the Brain. *J. Neurosci. Off. J. Soc. Neurosci.* **2015**, *35*, 6575–6583, doi:10.1523/JNEUROSCI.4298-14.2015.
7. Nath, A.; Schwartz, G.W. Cardinal Orientation Selectivity Is Represented by Two Distinct Ganglion Cell Types in Mouse Retina. *J. Neurosci. Off. J. Soc. Neurosci.* **2016**, *36*, 3208–3221, doi:10.1523/JNEUROSCI.4554-15.2016.
8. Schmidt, T.M.; Kofuji, P. Functional and Morphological Differences among Intrinsically Photosensitive Retinal Ganglion Cells. *J. Neurosci. Off. J. Soc. Neurosci.* **2009**, *29*, 476–482, doi:10.1523/JNEUROSCI.4117-08.2009.
9. Schmidt, T.M.; Kofuji, P. Structure and Function of Bistratified Intrinsically Photosensitive Retinal Ganglion Cells in the Mouse. *J. Comp. Neurol.* **2011**, *519*, 1492–1504, doi:10.1002/cne.22579.
10. Schmidt, T.M.; Kofuji, P. Differential Cone Pathway Influence on Intrinsically Photosensitive Retinal Ganglion Cell Subtypes. *J. Neurosci. Off. J. Soc. Neurosci.* **2010**, *30*, 16262–16271, doi:10.1523/JNEUROSCI.3656-10.2010.
11. Dumitrescu, O.N.; Pucci, F.G.; Wong, K.Y.; Berson, D.M. Ectopic Retinal ON Bipolar Cell Synapses in the OFF Inner Plexiform Layer: Contacts with Dopaminergic Amacrine Cells

and Melanopsin Ganglion Cells. *J. Comp. Neurol.* **2009**, *517*, 226–244, doi:10.1002/cne.22158.
12. Hattar, S.; Kumar, M.; Park, A.; Tong, P.; Tung, J.; Yau, K.-W.; Berson, D.M. Central Projections of Melanopsin-Expressing Retinal Ganglion Cells in the Mouse. *J. Comp. Neurol.* **2006**, *497*, 326–349, doi:10.1002/cne.20970.
13. Rivlin-Etzion, M.; Wei, W.; Feller, M.B. Visual Stimulation Reverses the Directional Preference of Direction-Selective Retinal Ganglion Cells. *Neuron* **2012**, *76*, 518–525, doi:10.1016/j.neuron.2012.08.041.
14. Estevez, M.E.; Fogerson, P.M.; Ilardi, M.C.; Borghuis, B.G.; Chan, E.; Weng, S.; Auferkorte, O.N.; Demb, J.B.; Berson, D.M. Form and Function of the M4 Cell, an Intrinsically Photosensitive Retinal Ganglion Cell Type Contributing to Geniculocortical Vision. *J. Neurosci. Off. J. Soc. Neurosci.* **2012**, *32*, 13608–13620, doi:10.1523/JNEUROSCI.1422-12.2012.
15. Quattrochi, L.E.; Stabio, M.E.; Kim, I.; Ilardi, M.C.; Michelle Fogerson, P.; Leyrer, M.L.; Berson, D.M. The M6 Cell: A Small-Field Bistratified Photosensitive Retinal Ganglion Cell. *J. Comp. Neurol.* **2019**, *527*, 297–311, doi:10.1002/cne.24556.
16. Baver, S.B.; Pickard, G.E.; Sollars, P.J.; Pickard, G.E. Two Types of Melanopsin Retinal Ganglion Cell Differentially Innervate the Hypothalamic Suprachiasmatic Nucleus and the Olivary Pretectal Nucleus. *Eur. J. Neurosci.* **2008**, *27*, 1763–1770, doi:10.1111/j.1460-9568.2008.06149.x.
17. Stabio, M.E.; Sabbah, S.; Quattrochi, L.E.; Ilardi, M.C.; Fogerson, P.M.; Leyrer, M.L.; Kim, M.T.; Kim, I.; Schiel, M.; Renna, J.M.; et al. The M5 Cell: A Color-Opponent Intrinsically Photosensitive Retinal Ganglion Cell. *Neuron* **2018**, *97*, 150-163.e4, doi:10.1016/j.neuron.2017.11.030.
18. Zhao, X.; Stafford, B.K.; Godin, A.L.; King, W.M.; Wong, K.Y. Photoresponse Diversity among the Five Types of Intrinsically Photosensitive Retinal Ganglion Cells. *J. Physiol.* **2014**, *592*, 1619–1636, doi:10.1113/jphysiol.2013.262782.
19. Sonoda, T.; Okabe, Y.; Schmidt, T.M. Overlapping Morphological and Functional Properties between M4 and M5 Intrinsically Photosensitive Retinal Ganglion Cells. *J. Comp. Neurol.* **2020**, *528*, 1028–1040, doi:10.1002/cne.24806.
20. Viney, T.J.; Balint, K.; Hillier, D.; Siegert, S.; Boldogkoi, Z.; Enquist, L.W.; Meister, M.; Cepko, C.L.; Roska, B. Local Retinal Circuits of Melanopsin-Containing Ganglion Cells Identified by Transsynaptic Viral Tracing. *Curr. Biol. CB* **2007**, *17*, 981–988, doi:10.1016/j.cub.2007.04.058.
21. Krieger, B.; Qiao, M.; Rousso, D.L.; Sanes, J.R.; Meister, M. Four Alpha Ganglion Cell Types in Mouse Retina: Function, Structure, and Molecular Signatures. *PloS One* **2017**, *12*, e0180091, doi:10.1371/journal.pone.0180091.
22. Jacoby, J.; Schwartz, G.W. Three Small-Receptive-Field Ganglion Cells in the Mouse Retina Are Distinctly Tuned to Size, Speed, and Object Motion. *J. Neurosci. Off. J. Soc. Neurosci.* **2017**, *37*, 610–625, doi:10.1523/JNEUROSCI.2804-16.2016.
23. Pang, J.-J.; Gao, F.; Wu, S.M. Light-Evoked Excitatory and Inhibitory Synaptic Inputs to ON and OFF Alpha Ganglion Cells in the Mouse Retina. *J. Neurosci. Off. J. Soc. Neurosci.* **2003**, *23*, 6063–6073.
24. Huberman, A.D.; Manu, M.; Koch, S.M.; Susman, M.W.; Lutz, A.B.; Ullian, E.M.; Baccus, S.A.; Barres, B.A. Architecture and Activity-Mediated Refinement of Axonal Projections from a Mosaic of Genetically Identified Retinal Ganglion Cells. *Neuron* **2008**, *59*, 425–438, doi:10.1016/j.neuron.2008.07.018.
25. Rousso, D.L.; Qiao, M.; Kagan, R.D.; Yamagata, M.; Palmiter, R.D.; Sanes, J.R. Two Pairs of ON and OFF Retinal Ganglion Cells Are Defined by Intersectional Patterns of Transcription Factor Expression. *Cell Rep.* **2016**, *15*, 1930–1944, doi:10.1016/j.celrep.2016.04.069.

26. Johnson, K.P.; Zhao, L.; Kerschensteiner, D. A Pixel-Encoder Retinal Ganglion Cell with Spatially Offset Excitatory and Inhibitory Receptive Fields. *Cell Rep.* **2018**, *22*, 1462–1472, doi:10.1016/j.celrep.2018.01.037.
27. Laboissonniere, L.A.; Goetz, J.J.; Martin, G.M.; Bi, R.; Lund, T.J.S.; Ellson, L.; Lynch, M.R.; Mooney, B.; Wickham, H.; Liu, P.; et al. Molecular Signatures of Retinal Ganglion Cells Revealed through Single Cell Profiling. *Sci. Rep.* **2019**, *9*, 15778, doi:10.1038/s41598-019-52215-4.
28. Zhao, X.; Chen, H.; Liu, X.; Cang, J. Orientation-Selective Responses in the Mouse Lateral Geniculate Nucleus. *J. Neurosci. Off. J. Soc. Neurosci.* **2013**, *33*, 12751–12763, doi:10.1523/JNEUROSCI.0095-13.2013.
29. Baden, T.; Berens, P.; Franke, K.; Román Rosón, M.; Bethge, M.; Euler, T. The Functional Diversity of Retinal Ganglion Cells in the Mouse. *Nature* **2016**, *529*, 345–350, doi:10.1038/nature16468.
30. Martersteck, E.M.; Hirokawa, K.E.; Evarts, M.; Bernard, A.; Duan, X.; Li, Y.; Ng, L.; Oh, S.W.; Ouellette, B.; Royall, J.J.; et al. Diverse Central Projection Patterns of Retinal Ganglion Cells. *Cell Rep.* **2017**, *18*, 2058–2072, doi:10.1016/j.celrep.2017.01.075.
31. Harris, J.A.; Hirokawa, K.E.; Sorensen, S.A.; Gu, H.; Mills, M.; Ng, L.L.; Bohn, P.; Mortrud, M.; Ouellette, B.; Kidney, J.; et al. Anatomical Characterization of Cre Driver Mice for Neural Circuit Mapping and Manipulation. *Front. Neural Circuits* **2014**, *8*, 76, doi:10.3389/fncir.2014.00076.
32. Yonehara, K.; Shintani, T.; Suzuki, R.; Sakuta, H.; Takeuchi, Y.; Nakamura-Yonehara, K.; Noda, M. Expression of SPIG1 Reveals Development of a Retinal Ganglion Cell Subtype Projecting to the Medial Terminal Nucleus in the Mouse. *PloS One* **2008**, *3*, e1533, doi:10.1371/journal.pone.0001533.
33. Duan, X.; Qiao, M.; Bei, F.; Kim, I.-J.; He, Z.; Sanes, J.R. Subtype-Specific Regeneration of Retinal Ganglion Cells Following Axotomy: Effects of Osteopontin and MTOR Signaling. *Neuron* **2015**, *85*, 1244–1256, doi:10.1016/j.neuron.2015.02.017.
34. Zhu, Y.; Xu, J.; Hauswirth, W.W.; DeVries, S.H. Genetically Targeted Binary Labeling of Retinal Neurons. *J. Neurosci. Off. J. Soc. Neurosci.* **2014**, *34*, 7845–7861, doi:10.1523/JNEUROSCI.2960-13.2014.
35. Ivanova, E.; Lee, P.; Pan, Z.-H. Characterization of Multiple Bistratified Retinal Ganglion Cells in a Purkinje Cell Protein 2-Cre Transgenic Mouse Line. *J. Comp. Neurol.* **2013**, *521*, 2165–2180, doi:10.1002/cne.23279.
36. Tien, N.-W.; Pearson, J.T.; Heller, C.R.; Demas, J.; Kerschensteiner, D. Genetically Identified Suppressed-by-Contrast Retinal Ganglion Cells Reliably Signal Self-Generated Visual Stimuli. *J. Neurosci. Off. J. Soc. Neurosci.* **2015**, *35*, 10815–10820, doi:10.1523/JNEUROSCI.1521-15.2015.
37. Ecker, J.L.; Dumitrescu, O.N.; Wong, K.Y.; Alam, N.M.; Chen, S.-K.; LeGates, T.; Renna, J.M.; Prusky, G.T.; Berson, D.M.; Hattar, S. Melanopsin-Expressing Retinal Ganglion-Cell Photoreceptors: Cellular Diversity and Role in Pattern Vision. *Neuron* **2010**, *67*, 49–60, doi:10.1016/j.neuron.2010.05.023.
38. Fernandez, D.C.; Chang, Y.-T.; Hattar, S.; Chen, S.-K. Architecture of Retinal Projections to the Central Circadian Pacemaker. *Proc. Natl. Acad. Sci. U. S. A.* **2016**, *113*, 6047–6052, doi:10.1073/pnas.1523629113.
39. Wang, Q.; Yue, W.W.S.; Jiang, Z.; Xue, T.; Kang, S.H.; Bergles, D.E.; Mikoshiba, K.; Offermanns, S.; Yau, K.-W. Synergistic Signaling by Light and Acetylcholine in Mouse Iris Sphincter Muscle. *Curr. Biol. CB* **2017**, *27*, 1791-1800.e5, doi:10.1016/j.cub.2017.05.022.
40. Gong, S.; Zheng, C.; Doughty, M.L.; Losos, K.; Didkovsky, N.; Schambra, U.B.; Nowak, N.J.; Joyner, A.; Leblanc, G.; Hatten, M.E.; et al. A Gene Expression Atlas of the Central Nervous System Based on Bacterial Artificial Chromosomes. *Nature* **2003**, *425*, 917–925, doi:10.1038/nature02033.

41. Schmidt, T.M.; Taniguchi, K.; Kofuji, P. Intrinsic and Extrinsic Light Responses in Melanopsin-Expressing Ganglion Cells during Mouse Development. *J. Neurophysiol.* **2008**, *100*, 371–384, doi:10.1152/jn.00062.2008.
42. Jiang, Z.; Yue, W.W.S.; Chen, L.; Sheng, Y.; Yau, K.-W. Cyclic-Nucleotide- and HCN-Channel-Mediated Phototransduction in Intrinsically Photosensitive Retinal Ganglion Cells. *Cell* **2018**, *175*, 652-664.e12, doi:10.1016/j.cell.2018.08.055.
43. Do, M.T.H.; Kang, S.H.; Xue, T.; Zhong, H.; Liao, H.-W.; Bergles, D.E.; Yau, K.-W. Photon Capture and Signalling by Melanopsin Retinal Ganglion Cells. *Nature* **2009**, *457*, 281–287, doi:10.1038/nature07682.
44. Hattar, S.; Liao, H.W.; Takao, M.; Berson, D.M.; Yau, K.W. Melanopsin-Containing Retinal Ganglion Cells: Architecture, Projections, and Intrinsic Photosensitivity. *Science* **2002**, *295*, 1065–1070, doi:10.1126/science.1069609.
45. Kim, I.-J.; Zhang, Y.; Yamagata, M.; Meister, M.; Sanes, J.R. Molecular Identification of a Retinal Cell Type That Responds to Upward Motion. *Nature* **2008**, *452*, 478–482, doi:10.1038/nature06739.
46. Lu, Q.; Ivanova, E.; Ganjawala, T.H.; Pan, Z.-H. Cre-Mediated Recombination Efficiency and Transgene Expression Patterns of Three Retinal Bipolar Cell-Expressing Cre Transgenic Mouse Lines. *Mol. Vis.* **2013**, *19*, 1310–1320.
47. Münch, T.A.; da Silveira, R.A.; Siegert, S.; Viney, T.J.; Awatramani, G.B.; Roska, B. Approach Sensitivity in the Retina Processed by a Multifunctional Neural Circuit. *Nat. Neurosci.* **2009**, *12*, 1308–1316, doi:10.1038/nn.2389.
48. Zhang, Y.; Kim, I.-J.; Sanes, J.R.; Meister, M. The Most Numerous Ganglion Cell Type of the Mouse Retina Is a Selective Feature Detector. *Proc. Natl. Acad. Sci. U. S. A.* **2012**, *109*, E2391-2398, doi:10.1073/pnas.1211547109.
49. Sabbah, S.; Berg, D.; Papendorp, C.; Briggman, K.L.; Berson, D.M. A Cre Mouse Line for Probing Irradiance- and Direction-Encoding Retinal Networks. *eNeuro* **2017**, *4*, ENEURO.0065-17.2017, doi:10.1523/ENEURO.0065-17.2017.
50. Parmhans, N.; Sajgo, S.; Niu, J.; Luo, W.; Badea, T.C. Characterization of Retinal Ganglion Cell, Horizontal Cell, and Amacrine Cell Types Expressing the Neurotrophic Receptor Tyrosine Kinase Ret. *J. Comp. Neurol.* **2018**, *526*, 742–766, doi:10.1002/cne.24367.
51. Parmhans, N.; Fuller, A.D.; Nguyen, E.; Chuang, K.; Swygart, D.; Wienbar, S.R.; Lin, T.; Kozmik, Z.; Dong, L.; Schwartz, G.W.; et al. Identification of Retinal Ganglion Cell Types and Brain Nuclei Expressing the Transcription Factor Brn3c/Pou4f3 Using a Cre Recombinase Knock-in Allele. *J. Comp. Neurol.* **2021**, *529*, 1926–1953, doi:10.1002/cne.25065.
